# Exploring the reported adverse effects of COVID-19 vaccines among vaccinated Arab populations: a multi-national survey study

**DOI:** 10.1038/s41598-024-54886-0

**Published:** 2024-02-27

**Authors:** Samar A. Amer, Ali Al-Zahrani, Esraa A. Imam, Ehab M. Ishteiwy, Ines F. Djelleb, Lina R. Abdullh, Dana Ballaj, Youmna A. Amer, Rehab H. El-Sokkary, Arafa M. Elshabrawy, Georgette Eskander, Jaffer Shah, Muhammad Liaquat Raza, Abdulaziz Majed A. Aba ALsafa, Hossam Tharwat Ali, Hebatallah M. Fawzy

**Affiliations:** 1 Department of Public Health and Community Medicine, Faculty of Medicine, Zagzig University, Zagazig, Egypt; 2 Membership at the Royal Colleague of General Practitioners [INT], London, UK; 3 Department of Mental Health Primary Care, Nova University, Lisbon, Portugal; 4 Research and Innovation Group, King Faisal Specialist Hospital and Research Center, Riyadh, Saudi Arabia; 5 Department of Family Medicine, Faculty of Medicine, MOH, Riyadh, Saudi Arabia; 6 Internship Year at Albayda Medical Center (AMC), Omar-Almukhtar University, Albayad, Libya; 7 Faculty of Medicine, Badji Mokhtar, 23000 Annaba, Algeria; 8 Department of Dermatology, Faculty of Medicine, University of Baghdad, Baghdad, Iraq; 9 Faculty of Medicine, University of Aleppo, Aleppo, Syria; 10 Department of Rheumatology and Rehabilitation, Faculty of Medicine, Zagzig University, Zagazig, Egypt; 11 Medical Microbiology and Immunology, Faculty of Medicine, Zagazig University, Zagazig, Egypt; 12 Endocrinology and Diabetes Division, Department of Internal Medicine, Faculty of Medicine, Zagazig University, Zagazig, Egypt; 13 Department of Biochemistry, Faculty of Pharmacy, Ain Shams University, Cairo, Egypt; 14 Weill Cornell Medicine, New York, NY USA; 15 Al Fatima Hospital, Karachi, Pakistan; 16 Specialist Nursing, Ministry of Health, Riyadh, Saudi Arabia; 17 Qena Faculty of Medicine, South Valley University, Qena, 83621 Egypt; 18 Lecturer of Public Health and Community Medicine, Faculty of Medicine, Zagazig University, Zagazig, Egypt

**Keywords:** Adverse effects, SARS-CoV-2, COVID-19, Coronavirus, Vaccination, Arab Populations, Vaccines, Health care, Medical research, Signs and symptoms

## Abstract

The coronavirus disease 2019 (COVID-19) pandemic has been a major challenge worldwide for the past years with high morbidity and mortality rates. While vaccination was the cornerstone to control the pandemic and disease spread, concerns regarding safety and adverse events (AEs) have been raised lately. A cross-sectional study was conducted between January 1st and January 22nd, 2022, in six Arabic countries namely Saudi Arabia, Egypt, Syria, Libya, Iraq, and Algeria. We utilized a self-administered questionnaire validated in Arabic which encompassed two main parts. The first was regarding sociodemographic data while the second was about COVID-19 vaccination history, types, doses, and experienced AEs. A multistage sampling was employed in each country, involving the random selection of three governorates from each country, followed by the selection of one urban area and one rural area from each governorate. We included the responses of 1564 participants. The most common AEs after the first and second doses were local AEs (67.9% and 46.6%, respectively) followed by bone pain and myalgia (37.6% and 31.8%, respectively). After the third dose, the most common AEs were local AEs (45.7%) and fever (32.4%). Johnson and Johnson, Sputnik Light, and Moderna vaccines showed the highest frequency of AEs. Factors associated with AEs after the first dose included an increase in age (aOR of 61–75 years compared to the 12–18 years group: 2.60, 95% CI: 1.59–4.25, p = 0.001) and male gender (OR: 0.72, 95% CI: 0.63–0.82, p < 0.001). The cumulative post-vaccination COVID-19 disease was reported with Sinovac (16.1%), Sinopharm (15.8%), and Johnson and Johnson (14.9) vaccines. History of pre-vaccination SARS-CoV-2 infection significantly increases the risk of post-vaccination COVID-19 after the first, second, and booster doses (OR: 3.09, CI: 1.9–5.07, p < 0.0001; OR: 2.56, CI: 1.89–3.47, p < 0.0001; and OR: 2.94, CI: 1.6–5.39, p = 0.0005 respectively). In conclusion, AEs were common among our participants, especially local AEs. Further extensive studies are needed to generate more generalizable data regarding the safety of different vaccines.

## Introduction

The ongoing coronavirus disease 2019 (COVID-19) pandemic poses a significant global health challenge, with over 772 million confirmed cases and around seven million deaths as of 30 November 2023, according to the World Health Organization (WHO)^1^. Vaccination has emerged as the most promising intervention to control the pandemic^2^. By the beginning of 2021, numerous vaccine candidates had received emergency use authorization (EUA), and countries had preordered more than 10 billion vaccine doses by December 2020^3^. Globally, as of 22 November 2023, more than 13 billion vaccine doses have been administered^1^.

Although the standard timeline for vaccine development is 10–14 years, numerous COVID-19 vaccines have been created during this unusual period of accelerated clinical progress^4,5^. They have been developed using different technological platforms, including mRNA vaccines such as Pfizer-BioNTech and Moderna, adenovirus vector vaccines such as AstraZeneca, Sputnik V, and Janssen, and inactivated killed vaccines like Sinopharm^6–8^. The Centers for Disease Control (CDC) recommends COVID-19 vaccination for all eligible individuals, regardless of their status of previous infection with the severe acute respiratory syndrome coronavirus 2 (SARS-CoV-2), as vaccines have shown high efficacy in preventing hospitalization, severe illness, and death^2,9^.

However, vaccine hesitancy and refusal remain significant challenges that combat the vaccination process of different pandemics^2,10^. Regarding COVID-19, vaccination acceptance rates varied widely across countries, with rates of 69% in some regions but as low as 11% in others. This variability can be attributed to factors such as vaccine availability, mandatory vaccination policies, public knowledge and attitudes towards vaccines, perceived effectiveness and cost, and experience of adverse events following immunization (AEFI) with COVID-19 vaccines^2,11–14^. The WHO defined COVID-19 AEFI as "any untoward medical occurrence which follows immunization and which does not necessarily have a causal relationship with the usage of the vaccine". AEFI can be categorized into five groups: vaccine product-related events, vaccine quality-related events, immunization error-related events, immunization-induced stress, and coincidental category, which has no direct relationship with the vaccine or any of the above but occurs soon after vaccination and hence may be attributed to it nonetheless^9,15^.

Generally, vaccine adverse events (AEs) occur within six weeks of vaccination and typically resolve within a few days in both children and adults^14,16,17^. Therefore, monitoring the COVID-19 vaccine for eight weeks after the final dose is recommended. After a vaccine is licensed, the CDC and the United States Food and Drug Administration (FDA) utilize a passive reporting system named the Vaccination Adverse Event Reporting System (VAERS) to conduct post-licensure surveillance to collect, monitor, and analyze reports of post-COVID-19 vaccination AEs^9,15,18,19^. Health institutions should continuously monitor, and report AEs associated with vaccines and medications to the relevant authorities through healthcare professionals^20^. Although adequate safety and efficacy of vaccines are the key elements for their licensure, the fast-tracking processes of vaccine development may heighten the risk of increased AEs as some data may go missing or unnoticed due to the accelerated process^21,22^. The faster approvals in the case of the COVID-19 vaccine compared to the conventional vaccine approval process is another important reason for hesitancy^21^.

To enhance public acceptance of COVID-19 vaccines and establish trust in vaccine safety and understand potential adverse effects, clear and reliable information is essential. The National Institutes of Health (NIH) urged further research on the effects of COVID-19 vaccines^12^. Despite the WHO's call for high-quality research on the negative health, social, and economic effects of COVID-19 vaccines, conclusive evidence is still lacking, necessitating further investigation into the factors contributing to these effects^2,11,23^. Many observational studies were conducted to monitor or detect the AEs following COVID-19 vaccinations with a wide range of AE rates between 13 and 90%^12,24–33^. Fewer studies focusing only on one country, one province, or one area were performed in the Arab countries with most studies done in Saudi Arabia and Egypt^33–41^. Hence, this multicenter study aimed to explore the AEs of different types and doses of COVID-19 vaccines and identify associated factors among vaccinated participants in six Arabic countries.

The primary objective of this study was to investigate the reported adverse effects (AEs) associated with different types and doses of COVID-19 vaccines among vaccinated participants in six Arabic countries. The secondary objective was to determine the potential factors associated with AEs, including demographic factors such as age, gender, body mass index (BMI), pre-vaccination comorbidities, and previous SARS-CoV-2 infection. Additionally, we aimed to explore the cumulative incidence of post-vaccination COVID-19 with different vaccine types. Through comprehensive data analysis and exploration, this study aimed to contribute to the existing knowledge on the safety profile of COVID-19 vaccines, improve public acceptance, and facilitate evidence-based discussions and interventions surrounding vaccine acceptance and potential adverse effects.

## Results

### Demographic characteristics of the participants

Of the 1564 vaccinated participants, a total of 660 participants (42.2%) were between the ages of 19 and 30 years, 968 participants (61.9%) were females, 1286 participants (82.2%) had a university education or higher, and 1397 participants (89.3%) resided in urban areas. Additionally, 581 participants (37.1%) were housewives or unemployed, and Saudi Arabia had the highest response rate with 444 participants (28.4%). The mean BMI was found to be 30.2 ± 2.65 kg/m^2^ (Table 1).

**Table 1:** The sociodemographic date, and its relationship with the vaccine adverse effects.

Variables	Total (N = 1564) F (%) *	The AE after the first dose of COVID-19 vaccination			
		No AE (N = 335) F (%)	Local and /or general AEs (N = 1032) F (%)	Systemic, and/or serious AEs (N = 197) F (%)	P-value of χ^2^ test #
**Age (years)**					
12–18	57 (3.6)	6 (10.5)	46 (80.7)	5 (8.8)	
19–30	660 (42.2)	144(21.8)	435(65.9)	81(12.3)	
> 30–45	463 (29.6)	87 (18.8)	300 (64.8)	76 (16.4)	< 0.001
> 45–60	241 (15.4)	55 (23.0)	159 (66.0)	27 (11.2)	
61–75	112 (7.2)	26 (23.2)	80 (71.4)	6 (5.8)	
> 75	31 (2.0)	17 (54.8)	12 (38.7)	2 (6.5)	
**Sex**					
Female	968 (61.9)	148 (15.3)	669 (69.1)	151 (15.6)	< 0.001
Male	596 (38.1)	187 (31.4)	363 (60.9)	46 (7.7)	
**Education**					
Read, write /primary	44 (2.8)	17 (38.9)	25 (56.8)	2 (4.5)	
Secondary /high	234 (15.0)	62 (26.5)	145 (62.0)	27 (11.5)	0.006
University or above	1286 (82.2)	258 (19.9)	862 (67.0)	168 (13.1)	
**Occupation**					
Unemployed	581 (37.1)	129 (22.2)	380 (65.4)	72 (12.4)	
Medical field	547 (35.0)	112 (20.5)	370 (67.6)	60 (13.8)	0.83
Other fields	436 (27.9)	94 (21.6)	282 (64.7)	65 (11.9)	
**Residence**					
Inside city (urban)	1397 (89.3)	290 (20.8)	923 (66.4)	184 (12.8)	0.34
Outside city (rural)	167 (10.7)	45 (29.6)	104 (62.3)	18 (10.8)	
**BMI (kg/m2)**					
Mean ± SD	30.2 ± 2.65	(23.9 ± 9.4)a	(30.4 ± 7.9)b	(38.6 ± 7.8)	0.04 **##**
**Country**					
Saudi Arabia (SA)	444 (28.4)	55 (12.4)	307 (69.1)	82 (18.5)	
Egypt	345 (22.0)	84 (24.4)	214 (61.9)	47 (13.7)	
Libya	218 (14.0)	81 (37.2)	121 (55.5)	16 (7.3)	
Algeria	213 (13.0)	58 (27.5)	144 (67.6)	11 (5.2)	< 0.001
Iraq	183 (11.7)	36 (19.7)	126 (68.8)	21 (11.5)	
Syria	124 (7.9)	16 (12.9)	97 (78.2)	7 (8.9)	
Others**	37 (2.4)	5 (13.5)	23 ( 62.2)	9 (24.3)	

*Percentages of the total column are from the total of each dose. Otherwise, F (%) is calculated per column.

^#^P-value of χ2 chi square test. Significance level < 0.05.

^##^ANOVA (analysis of variance) test. Post-hoc test to show the least significance difference between the subgroups/ alphabetical letter of different symbols means a significant difference between groups.

** Others include 37 Sudan participants.

Regarding medical and medication history, the majority of vaccinated participants (70.0%) reported not taking any medications, and 1200 participants (76.7%) did not have any comorbidities. and 840 participants (53.7%) had no history of previous SARS-CoV-2 infection before vaccination. Out of the 510 participants (32.6%) who had experienced SARS-CoV-2 infection, 435 (85.3%) were managed at home. The majority of participants (72.9%) were vaccinated under mandatory vaccination policies while 889 (56.9%) received the vaccine in a vaccine center or hospital (Table 2).

**Table 2:** Medical, medication and vaccination history and their relationships with the vaccine adverse effects.

History	Total (N = 1564) F (%) *	The AE after the first dose of COVID-19 vaccination			P-value of χ2 test #
		No AE (N = 335) F (%)	Local and /or general AEs (N = 1032) F (%)	Systemic, and/or serious AEs (N = 197) F (%)	
**Co-morbidities**					
No	1200 (76.7)	254 (21.2)	797 (66.4)	149 (12.4)	
Psychiatric/neurol**o**gi**cal**	60 (3.8)	5 (8.3)	38 (63.3)	17 (28.3)	0.003
Organic	291 (18.6)	73 (25.3)	187 (64.9)	28 (9.7)	
Both	13 (0.8)	3 (23.1)	9 (69.2)	1 (7.7)	
**Drug intake**					
None of the below	1095 (70.0)	234 (21.4)	739 (67.5)	122 (11.1)	
Supplements	274 (17.5)	51 (18.6)	166 (60.9)	57 (20.8)	0.004
Hormonal therapy	104 (6.7)	26 (25.0)	68 (65.4)	10 (19.6)	
Immune suppression medications	40 (2.6)	8 (20.0)	27 (67.5)	5 (12.5)	
Anti-coagulants	51 (3.3)	16 (31.4)	32 (62.7)	3 (5.9)	
**SARS-CoV-2 infection before vaccination**					
No	840 (53.7)	133 (15.8)	594 (70.7)	113 (13.5)	
Suspicious (symptoms)	214 (13.7)	110 (51.4)	89 (41.6)	15 (7.0)	
Yes, without symptoms	56 (3.6)	13 (23.3)	35 (62.2)	8 (14.3)	< 0.001
Yes, managed at home	435 (27.8)	78 (18.1)	229 (68.9)	57 (13.1)	
Yes, managed at hospitals	19 (1.2)	1 (5.3)	15 (78.9)	3 (15.8)	
**COVID-19 vaccination uptake**					
Mandatory	1136 (72.9)	248 (21.8)	766 (67.4)	122 (10.7)	0.74
My choice	428 ( 27.1)	87 (20.3)	266 (62.1)	75 (17.5)	
**Setting of COVID-19 vaccination uptake**					
Mobile campaign	98 (6.2)	35 (35.7)	50 (51.0)	13 (13.3)	< 0.001
Primary health care centers	577 (36.9)	141 (15.9)	362 (62.7)	56 (9.7)	
Vaccine center or hospital	889 (56.9)	159 (27.9)	620 (69.7)	128 (14.0)	

*Percentages of the total column are from the total of each dose. Otherwise, F (%) is calculated per column.

^#^P-value of χ2 chi square test. Significance level < 0.05.

**Figure 1:**
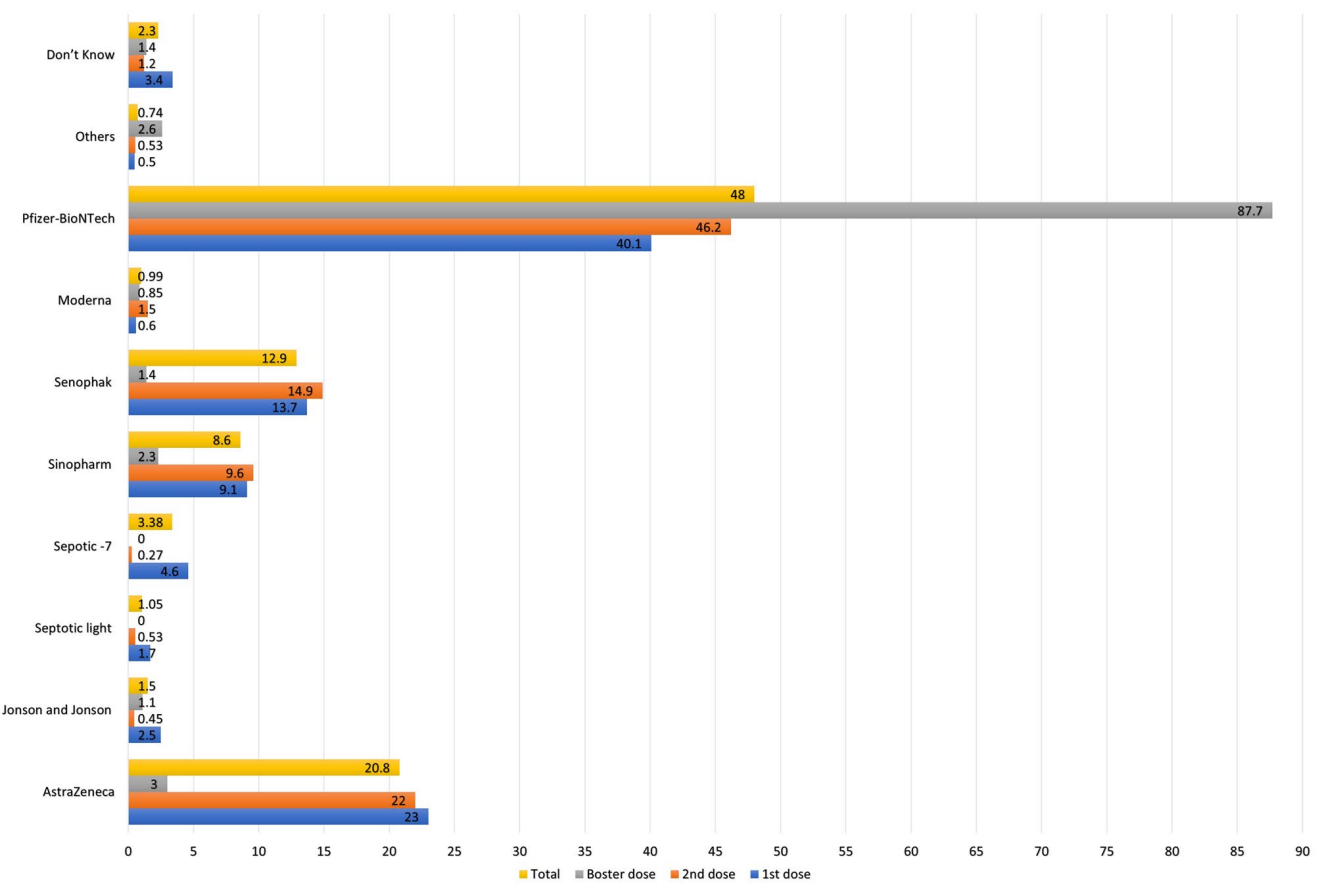
The relative frequency distribution of different types of vaccinations at different doses.

Out of the total 1564 participants who received the first dose of the COVID-19 vaccine, 1325 (84.7%) completed the recommended two-dose regimen, while only 350 (22.4%) received the booster dose. In terms of vaccine types, out of the total 3239 vaccination doses administered, Pfizer-BioNTech was the most common, given in 1551 doses (48.0%), followed by AstraZeneca in 672 doses (20.8%), and Sinovac in 416 doses (12.9%). The percentages of each vaccine type at different doses among our participants are shown in Fig. 1.

### Adverse effects post-COVID-19 vaccination

**Figure 2:**
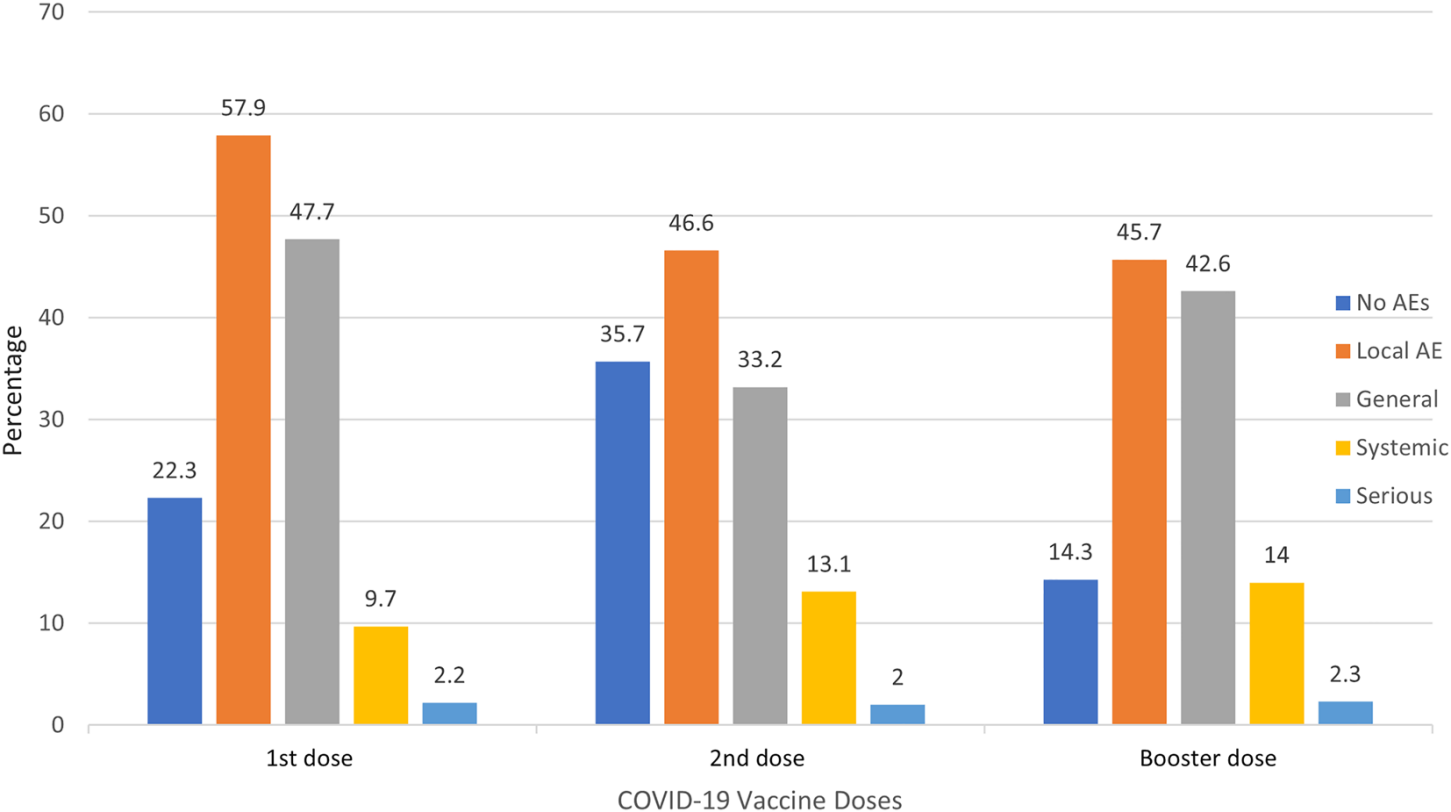
The distribution of AEs after each vaccination dose.

Overall, 1212 (77.7%) participants experienced an adverse event after the first COVID-19 dose, whereas 852 (64.3%) and 300 (85.7%) individuals had an adverse event following the second and booster doses respectively. For each dose, local AEs were the most experienced followed by general, systemic, and serious AEs (Fig. 2).

**Figure 3:**
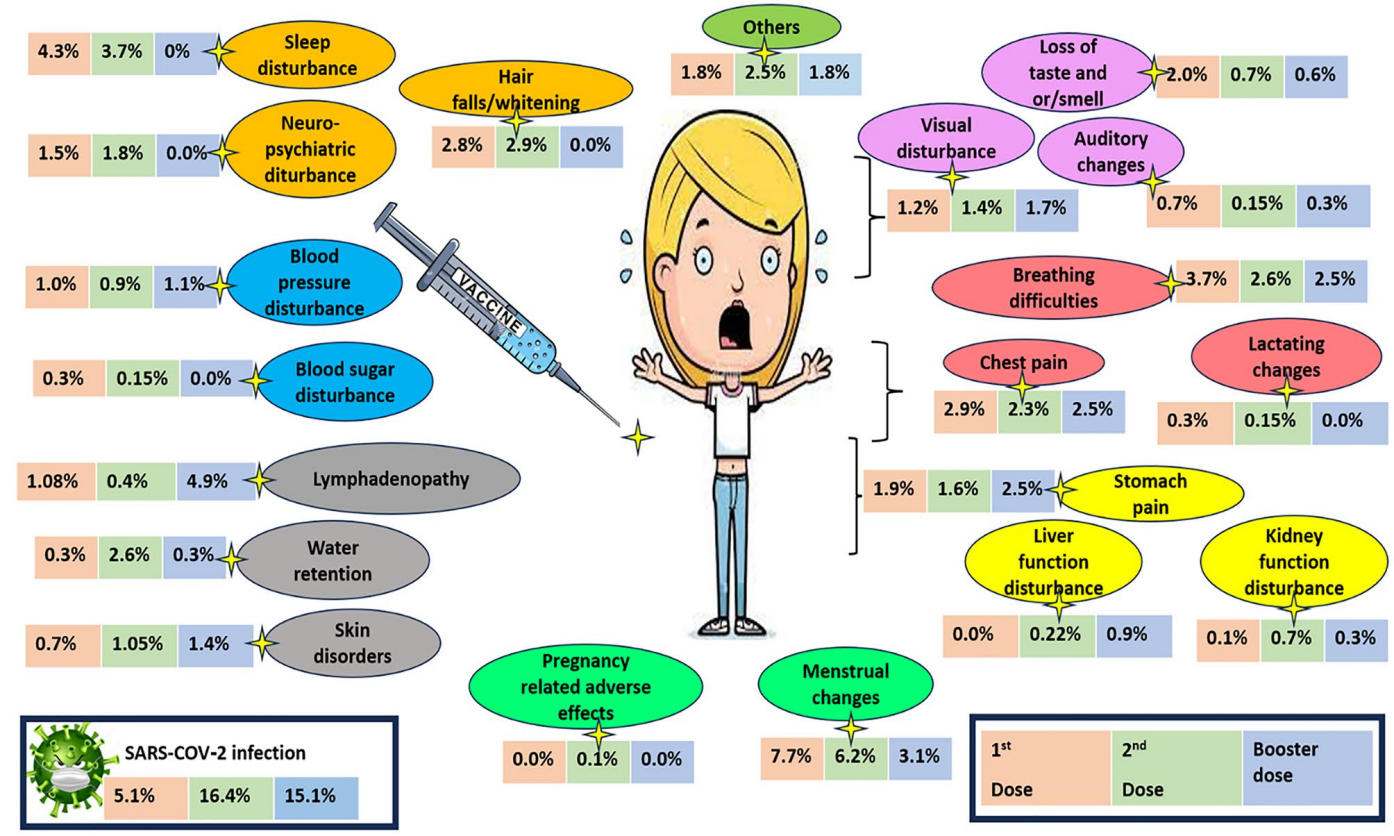
The details of AEs and SARS-COV-2 infection after each vaccination dose.

Regarding the occurrence of adverse effects (AEs) following COVID-19 vaccination, the most common AEs after the first dose were local AEs (57.9%) and fatigue (37.6%). After the second dose, the most common AEs were local AEs (46.6%) and fatigue (31.8%). Following the booster dose, the common AEs reported were local AEs in 160 participants (45.7%), fever in 82 participants (32.4%), and fatigue in 102 participants (30.0%). Approximately half of these AEs required no medical intervention, around half necessitated medical symptomatic treatment, and around 1% resulted in hospital admission. The details of the AEs among our participants are shown in Table 3 and Fig. 3.

**Table 3:** Adverse effects after COVID-19 vaccinations, and the required management.

Adverse events (AEs)	1st dose T = 1564 F (%)	2nd dose T = 1325 F (%)	Booster dose ## T = 350 F (%)
Local adverse events (AEs) at the injection site	906 (57.69)	617 617 (46.6)	160 160 (45.7)
**General constitutional AEs**			
Fever /chills	**523 (33.4)**	**401 (30.3)**	82 (32.4)
Nausea/vomiting	100 (6.3)	68 (5.1)	26 (7.4)
Body aches	**531 (34.1)**	**387 (29.2)**	**96 (27.4)**
Bone pain and myalgia	358 (22.8)	**311 (23.5)**	**82 (23.4)**
Fatigue	**588 (37.6)**	**421 (31.8)**	**102 (30.0)**
Headache	**378 (24.3)**	**259 (19.5)**	61 (17.4)
Running nose	63 (3.8)	34 (2.6)	7 (2.0)
**Systemic AEs**			
Water retention	5 (0.3)	34 (2.6)	1 (0.3)
Menstrual changes (T = 965) *	**75 (7.7)**	**53 (6.2)**	**11 (3.1)**
Chest pain	48 (2.9)	31 (2.3)	**9 (2.5)**
Breathing difficulties	**59 (3.7)**	35 (2.6)	**9 (2.5)**
Stomach pain (persistence)	30 (1.9)	21 (1.6)	**9 (2.5)**
Visual disturbance	20 (1.2)	19 (1.4)	6 (1.7)
Neuro-psychiatric disturbance	25 (1.5)	24 (1.8)	0 (0.0)
Blood pressure disturbance	18 (1.0)	12 (0.9)	4 (1.1)
Blood sugar disturbance	6 (0.3)	2 (0.15)	0 (0.0)
Liver function disturbance	0 (0.0)	3 (0.22)	3 (0.9)
Kidney function disturbance	3 (0.1)	9 (0.7)	1 (0.3)
Pregnancy changes (T = 965)*	0 (0.0)	1 (0.1)	0 (0.0)
Lactating changes (T = 965) *	3 (0.3)	2 (0.15)	0 (0.0)
Lymphadenopathy	17 (1.08)	6 (0.4)	**17 (4.9)**
Hair falls /whitening	**45 (2.8)**	39 (2.9)	0 (0.0)
Skin disorders	12 (0.7)	14 (1.05)	5 (1.4)
Loss of taste and or /smell	**32 (2.0)**	9 (0.7)	2 (0.6)
Auditory changes	11 (0.7)	2 (0.15)	1 (0.3)
Sleep disturbance	**69 (4.3)**	**49 (3.7)**	0 (0.0)
Others	20 (1.8)	33 (2.5)	18 (5.1)
**Serious AEs**			
Thrombosis	5 (0.3)	5 (0.4)	0 (0.0)
Convulsions	5 (0.3)	5 (0.4)	5 (1.4)
Thrombocytopenia **#**	5 (0.3)	7 (0.5)	0 (0.0)
Cardiac side effects (carditis/ arrhythmia)	18 (1.1)	3 (0.22)	3 (0.85)
Auto-immune diseases	11 (0.7)	18 (1.35)	0 (0.0)
Gillian-Barrie syndrome	1 (0.07)	0 (0.0)	0 (0.0)
Hypersensitivity	29 (1.7)	17 (1.3)	5 (1.4)
**Management of the adverse effects**			
Nothing	757 (48.4)	750 (56.6)	139 (39.7)
Medical symptomatic treatment	785 (50.2)	563 (42.5)	177 (50.6)
Medical consultation	57 (3.6)	42 (3.2)	15 (4.3)
Hospital admission	7 (0.5)	9 (0.67)	4 (1.1)
Home rest /sick leave from work	99 (6.3)	131 (9.9)	32 (9.1)

**#**Vaccine-induced immune thrombotic thrombocytopenia (VITT).

^##^Because the vaccination strategies regarding giving booster doses have not been made available in five countries only available in SA, the participants of these five countries should not be taken into consideration when evaluating the third dose.

F: Frequency; calculated per row.

*Total number of married women only = 965 females.

#### Adverse effects among different vaccine types

The frequency of AEs varied depending on the vaccine type. Among the vaccines, Johnson and Johnson (93.9%), Moderna (84.4%), Sputnik Light (85.3%), Sputnik V (82.6%), and Pfizer-BioNTech (82.1%) had significantly the highest frequency of reported AEs (P-value < 0.001). After the first dose, Johnson and Johnson had significantly the highest frequency of AEs (97.4%) while after the second dose, Pfizer-BioNTech (81.5%) and Sputnik V (80.6%) had the highest AEs rate. The details of the AE rate distribution based on vaccine type are described in Table 4.

**Table 4:** The relationship between the reported AEs after COVID-19 vaccinations, and the type of vaccine.

	AstraZeneca	J&J	Sputnik V	Sputnik-light	Sinovac	Sinopharm	Moderna	Pfizer	P of Fisher’s exact test *
	F (%)	F (%)	F (%)	F (%)	F (%)	F (%)	F (%)	F (%)	
**(a) The first dose (T = 1564)**									
No AEs	38 (10.4)	1 (2.6)	12 (16.4)	3 (11.1)	71 (33.0)	69 (48.3)	1 (11.1)	107 (17.0)	< 0.001
Local AEs only	31 (8.5)	3 (7.7)	13 (17.8)	5 (18.5)	58 (27.0)	28 (19.6)	1 (11.1)	121 (19.2)	
Local + general AEs	243 (66.4)	29 (74.4)	39 (53.4)	17 (63.0)	75 (34.9)	34 (23.8)	5 (55.6)	304 (48.2)	
Local + systemic AEs	44 (12.0)	4 (10.3)	7 (9.6)	2 (7.4)	9 (4.2)	10 (7.0)	2 (22.2)	83 (13.3)	
Serious AEs	10 (2.7)	3 (7.7)	2 (2.7)	0 (0.0)	2 (0.9)	2 (1.4)	0 (0.0)	16 (2.5)	
**(b) The 2**nd** dose ( T = 1325)**									
No AEs	106 (35.7)	2 (33.3)	7 (19.4)	2 (28.6)	81 (63.8)	115 (58.4)	4 (20.0)	133 (18.5)	< 0.001
Local AEs only	33 (11.1)	0 (0.0)	4 (11.1)	0 (0.0)	22 (17.3)	48 (24.4)	2 (10.0)	122 (19.9)	
Local + general AEs	123 (41.4)	3 (50.0)	20 (55.6)	3 (42.9)	16 (12.6)	20 (10.2)	7 (35.0)	248 (40.5)	
Local + systemic AEs	27 (9.1)	0 (0.0)	5 (13.9)	2 (28.6)	8 (6.3)	11 (5.6)	6 (30.0)	115 (18.8)	
Serious AEs	8 (2.7)	1 (16.7)	0 (0.0)	0 (0.0)	0 (0.0)	3 (1.5)	1 (5.0)	14 (2.3)	
**(c) The booster dose (T = 350)**									
No AEs	3 (33.3)	0 (0.0)	0 (0.0)	0 (0.0)	2 (50.0)	5 (62.5)	0 (0.0)	38 (12.6)	0.01
Local AEs only	0 (0.0)	2 (50.0)	0 (0.0)	0 (0.0)	2 (50.0)	2 (25.0)	3 (100.0)	67 (22.2)	
Local + general AEs	6 (66.7)	2 (50.0)	0 (0.0)	0 (0.0)	0 (0.0)	1 (12.5)	0 (0.0)	140 (46.4)	
Local + systemic AEs	0 (0.0)	0 (0.0)	0 (0.0)	0 (0.0)	0 (0.0)	0 (0.0)	0 (0.0)	49 (16.2)	
Serious AEs	0 (0.0)	0 (0.0)	0 (0.0)	0 (0.0)	0 (0.0)	0 (0.0)	0 (0.0)	8 (2.6)	

Total number of doses									
	N = 672	N = 49	N = 109	N = 34	N = 416	N = 278	N = 32	N = 1551	
No AE	288 (42.9)	3 (6.1)	19 (17.4)	5 (14.7)	154 (37.0)	189 (67.9)	5 (15.6)	278 (17.9)	< 0.001
AEs	384 (57.1)	46 (93.9)	90 (82.6)	29 (85.3)	262 (62.9)	89 (32.1)	27 ( 84.4)	1273 (82.1)	

F: Frequency; calculated per column.

* Fisher’s exact test; P-value significance < 0.05.

#### Determinants of adverse effects following the first dose of the COVID-19 vaccine

In terms of sociodemographic characteristics, a statistically significant association was found between reported AEs and certain parameters. AEs were more common among females (84.7%), and individuals with a higher education level (79.1%). Libyan participants had the lowest occurrence of AEs (62.8%) (Table 1). Participants using anticoagulants (31.4%), individuals with previous SARS-CoV-2 infection (18.04%), and those vaccinated through mobile campaigns (35.7%) were associated with lower rates of AE reports (Table 2).

Univariate analysis revealed that old age, female sex, nationality, obesity, and comorbidities were statistically significant factors associated with AEs after the first dose of the COVID-19 vaccination (p < 0.05). The use of supplements was found to be a protective factor (OR: 0.56, 95% CI: [0.41–0.76], p = 0.012). Following the multivariable model, independent associated factors of AEs following the first dose of COVID-19 vaccination were age, gender, and certain comorbidities. Higher ages significantly increase the odds of having AEs with the over-75-year group having the highest odds (aOR: 2.71, 95% CI: 1.59–4.42, p < 0.001. Males were less likely to have AEs compared to females (aOR: 0.72, 95% CI: 0.63–0.82, p < 0.001). The details of the regression analysis of factors determining the odds of experiencing AEs are described in Table 5.

**Table 5:** Factors associated with the COVID-19 vaccine adverse effects after the first dose.

Variables	Univariable analyses		Multiple logistic regression model	
Demographics:	OR (95% CI)	P-value *	aOR (95% CI)	P-value *
**Age (y)**				
12–18 **(Reference)**	–	–	–	–
19–30	1.67 (1.07–2.62)	0.025	1.73 (1.06–2.82)	0.028
31–45	1.71 (1.19–2.73)	0.007	1.77 (1.13–2.80)	0.014
46–60	2.20 (1.43–3.38)	< 0.001	2.16 (1.36–3.45)	0.001
61–75	2.68 (1.71–4.20)	< 0.001	2.60 (1.59–4.25)	< 0.001
> 75	2.71 (1.75–5.1)	< 0.001	2.71 (1.59–4.42)	< 0.001
**Sex**	0.55 (0.48–0.61)		0.72 (0.63–0.82)	
Female **(Reference)**				
Male		< 0.001		< 0.001
**Healthcare worker**			–	
No **(Reference)**	–	–	0.93 (0.79–1.10)	–
Yes	0.83 (0.72–0.97)	0.023		0.392
**Drug intake**				
None of the below **(Reference)**	–	–	–	–
Supplements	1.22 (1.00–1.49)	0.05	–	–
Anti-coagulants	0.56 (0.41–0.76)	0.012	–	–
Immune suppression medications	1.01 (0.60–1.69)	0.975	–	–
Hormonal therapy	0.47 (0.20–1.19)	0.117	–	–
**Co-morbidities**				
No **(Reference)**	–	–	–	–
Psychiatric /neurological	0.51 (0.42–0.62)	< 0.001	0.36 (0.22–0.61)	< 0.001
Organic	0.56 (0.41–0.76)	< 0.001	0.45 (0.19–1.09)	0.076
Both	1.23 (1.05–1.45)	< 0.001	2.68 (1.71–4.20)	< 0.001
**SARS-CoV-2 infection before vaccination**				
No **(Reference)**				
Suspicious (symptoms)	–	–		
Yes, without symptoms	0.57 (0.37–0.89)	0.014	–	–
Yes, managed at home	0.79 (0.37–1.68)	0.537	–	–
Yes, managed at hospitals	0.24 (0.12–0.50)	< 0.001	–	–
	0.72 (0.41–1.26)	0.25	–	–

*Statistically significant with at least 5% of the significance level.

(OR): Odds Ratio, (aOR): adjusted OR, (CI): Confidence Interval.

### Post-vaccination COVID-19 infection

#### Post-vaccination COVID-19 infection among different types of vaccines

Regarding vaccine types, the highest frequency of COVID-19 cases after the first dose was reported with the Johnson and Johnson vaccine (17.9%), Sputnik light vaccine (14.8%), and AstraZeneca vaccine (7.1%). After the second dose, Sinovac (32.5%), Sinopharm (25.4%), and Sputnik V (22.2%) had the highest frequency of COVID-19 cases. Overall, Sinovac (16.1%) followed by Sinopharm (15.8%) and Johnson and Johnson vaccines (14.2%) had the highest rate of post-vaccine COVID-19 (Table 6).

**Table 6:** COVID-19 after vaccination, and the type of COVID-19 vaccine.

Post-vaccination COVID-19	Astra-Zeneca F (%)	J&J# F (%)	Sputnik V F (%)	Sputnik-light F (%)	Sinovac F (%)	Sinophar-m F (%)	Moder-na F (%)	Pfizer-BioNTech F (%)	P of fisher exact t test *
**(a) The first dose (T = 1564)**									
No infection	340 (92.9)	32 (82.1)	68 (93.2)	23 (85.2)	210 (97.7)	133 (93.0)	9 (100.0)	609 (96.5)	< 0.001
Asymptomatic infection	3 (0.8)	2 (5.1)	2 (2.8)	0 (0.0)	2 (0.9)	3 (2.1)	0 (0.0)	4 (0.6)	
Symptomatic managed at home	21 (5.7)	5 (12.8)	3 (4.1)	4 (14.8)	3 (1.4)	7 (4.9)	0 (0.0)	18 (2.9)	
Symptomatic managed at the hospital	2 (0.5)	0 (0.0)	0 (0.0)	0 (0.0)	0 (0.0)	0 (0.0)	0 (0.0)	0 (0.0)	
**(b) The 2nd dose (T = 1325)**									
No infection	262 (93.9)	6 (100.0)	28 (77.8)	7 (100.0)	135 (67.5)	95 (74.6)	17 (85.0)	570 (93.1)	< 0.001
Asymptomatic infection	6 (2.1)	0 (0.0)	1 (1.6)	0 (0.0)	5 (2.6)	4 (3.2)	3 (15.0)	7 (1.1)	
Symptomatic managed at home	27 (9.3)	0 (0.0)	6 (16.7)	0 (0.0)	50 (26.2)	26 (20.6)	0 (0.0)	33 (5.4)	
Symptomatic managed at the hospital	2 (0.7)	0 (0.0)	1 (1.6)	0 (0.0)	7 (3.7)	2 (1.6)	0 (0.0)	2 (0.3)	
**(c) The booster dose (T = 350)**									
No infection	9 (100.0)	8 (100.0)	0 (0.0)	0 (0.0)	4 (100.0)	6 (80.0)	3 (100.0)	284 (92.2)	< 0.001
Asymptomatic infection	0 (0.0)	0 (0.0)	0 (0.0)	0 (0.0)	0 (0.0)	2 (20.0)	0 (0.0)	2 (0.6)	
Symptomatic managed at home	0 (0.0)	0 (0.0)	0 (0.0)	0 (0.0)	0 (0.0)	0 (0.0)	0 (0.0)	15 (4.9)	
Symptomatic managed at the hospital	0 (0.0)	0 (0.0)	0 (0.0)	0 (0.0)	0 (0.0)	0 (0.0)	0 (0.0)	7 (2.3)	

Total number of doses									
	T = 672	T = 49	T = 109	T = 34	T = 416	T = 278	T = 32	T = 1551	
No infection	611 (90.9)	42 (85.7)	96 (88.1)	30 (88.2)	349 (83.9)	234 (84.2)	24 (90.6)	1463 (94.3)	< 0.001
Infection	61 ( 9.1)	7 (14.2)	13 (11.9)	4 (11.8)	67 (16.1)	44 (15.8)	8 (9.4)	88 (5.7)	

*P < 0.05 there was statistically significant difference between different types of vaccines.

F frequency (calculated per row).

^##^ only single dose is required from J and Johnson vaccines, but due to unavailability, documentation, and restriction causes some participants received it as a second dose or booster dose.

#### Post-vaccination COVID-19 and its relationship with pre-vaccination COVID-19 infection

The frequency of post-vaccination COVID-19 cases was significantly associated with the pre-vaccination SARS-CoV-2 infection status. Among participants with no previous infection, the highest occurrence of COVID-19 cases was observed after the booster dose (12%), followed by the second dose (10.9%), followed by the first dose (2.7%). In contrast, among participants with a previous infection, the highest occurrence of COVID-19 cases was observed after the second dose (23.4%), followed by the booster dose (23.2) and the first dose (8%). Overall, having a pre-vaccination history of COVID-19 increases the risks of post-vaccination SARS-CoV-2 infection (Table 7).

**Table 7:** The relationship between the SARS-COV-2 infection prior to vaccination and post-vaccination COVID-19.

Variable	Total F (%) *	No past history of SARS-COV-2 infection F (%)	Past history of SARS-COV-2 infection F (%)	*P-value #*	OR [95% CI] *(P*) ##
**First dose (T** = **1564)**					
No	1483 (94.8)	817 (97.3)	666 (92.0)		
Yes, asymptomatic	17 (1.1)	2 (0.2)	15 (2.1)	< 0.001	3.09 [1.9–5.07]
Yes, symptomatic managed at home	62 (4.0)	20 (2.4)	42 (5.8)		(< 0.0001)
Yes, symptomatic managed at hospital	2 (0.1)	1 (0.1)	1 (0.1)		
**Second dose (T = 1325)**					
No	1114 (83.5)	644 (89.1)	480 (76.6)		
Yes, asymptomatic	50 (4.3)	16 (2.2)	34 (5.9)	< 0.001	2.56 [1.89–3.47]
Yes, symptomatic managed at home	146 (11.0)	57 (7.9)	89 (15.6)		(< 0.0001)
Yes, symptomatic managed at hospital	15 (1.1)	4 (0.8)	11 (1.9)		
**Booster dose (T = 350)**					
No	297 (84.9)	214 (88.0)	83 (76.8)		2.94 [1.6–5.39]
Yes, asymptomatic	33 (9.4)	15 (6.4)	18 (16.7)	0.09	− 0.0005
Yes, symptomatic managed at home	13 (3.7)	7 (3.0)	6 (6.4)		
Yes, symptomatic managed at hospital	7 (2.0)	6 (2.6)	1 (0.1)		

*Percentages of the total column are from the total of each dose. Otherwise, F (%) are calculated per column.

^*#*^* P-value* of χ2 chi square test. Significance level < 0.05.

^##^ No past history of SARS-COV-2 infection is the reference.

## Discussion

Since the beginning of the COVID-19 pandemic, the focus of research has primarily been on COVID-19 symptoms and vaccinations. Despite the widespread administration of millions of vaccine doses worldwide, concerns about the safety and efficacy of vaccinations continue to be raised. To address this, our study aimed to investigate the adverse events (AEs) associated with different types and doses of COVID-19 vaccines across six Arabic countries during the fourth wave of the pandemic.

The variation in the number of vaccinated participants among the studied Arab countries reflects differences in vaccine availability and compulsory vaccine regulations. For example, Saudi Arabia initiated vaccination for children aged 12 and older in July 2021 and mandated that all citizens and residents receive a booster dose by February 2022. In contrast, compulsory vaccination policies and booster doses had not been implemented in the remaining five countries at the time of data collection^46–48^.

The pattern of AEs after each dose aligns with previous reports^49^. This may be attributed to the cumulative immunological effect of the second dose rather than a direct immunological response^50^. We observed a lower frequency of AEs after the second dose with many types of vaccines compared to the first dose. However, we reported an increase in the frequency of AEs after the Sputnik V vaccine, local AEs after the Sinopharm vaccine, systemic AEs after the Pfizer-BioNTech vaccine, and serious AEs after the Johnson & Johnson (J&J) vaccine. Previous studies have shown different trends, with higher local and systemic AEs reported after the second dose of Pfizer-BioNTech and AstraZeneca vaccines^26,50–52^.

In our study, the most prevalent local AEs, such as pain, redness, and swelling at the injection site, were reported after the Pfizer-BioNTech, AstraZeneca, and Sinopharm vaccines. Previous studies conducted in the reported varying percentages were reported after the first and second doses^20,26,53^. The most commonly reported general AEs were fatigue, body aches, fever, headache, and myalgia, which is in line with published studies^20,49^.

Headache was reported in more than 50% of participants after the AstraZeneca vaccine^54–56^. There are no details about the pathophysiologic mechanisms, whether the intracellularly synthesized spike protein is produced by using mRNA vaccines, or the protein triggers the immune response from activated anti-inflammatory mediators such as prostaglandins, nitric oxide, and cytokines. Headache is the leading symptom of cerebrovascular thrombosis (CVT), including vaccine-induced ones. So, it's important to distinguish between vaccine-induced headaches and those caused by cerebrovascular thrombosis^54–56^.

Visual disturbances were reported by a small number of participants. There are reported cases of transient loss in the visual field due to possible acute vasospasm of the artery in the postchiasmatic visual pathway, triggered by the COVID-19 vaccine that resolved after two hours^57^. In other cases, macular detachment and severe choroidal thickening were detected causing visual loss and suggesting a potential inflammatory or autoimmune response to the vaccine^58–60^.

Elevations in blood pressure were observed among some vaccinated participants, which is consistent with reports of blood pressure surges after mRNA vaccines and an increase in home blood pressure after the first mRNA vaccine dose. Some patients required modification of anti-hypertensive drugs. This may be attributed to nervousness or white-coat hypertension. However there was no baseline data, and BP follow-up over a long period after vaccination is very important^56,61^.

Menstrual changes were reported among vaccinated females and it is noteworthy that by September 2, 2021, over 30,000 COVID-19-vaccinated females had reported menstrual changes to the United Kingdom’s Medicines and Healthcare Products Regulatory Agency (MHRA) Yellow Card surveillance system^12,62^. This might be a result of immunological effects on the hormones that regulate the menstrual cycle or biological effects of immune cells on the uterus lining, which contribute to the tissue's cyclical building and breaking down^12,63^.

Rheumatological symptoms such as bone pain, myalgia, body aches, and weariness were reported in our study, similar to some studies conducted in Italy, Libya, Iran, China, and Turkey^61,63–67^. These symptoms might be attributed to the immune response triggered by the vaccine, leading to transient inflammation and musculoskeletal discomfort^26,68^. It is important to note that these symptoms are generally self-limiting and resolve within a few days after vaccination. The association between COVID-19 vaccination and the occurrence of certain symptoms remains uncertain when compared to other vaccines. The hyper-inflammatory response triggered by the COVID-19 vaccine raises concerns about its potential as a risk factor for inflammatory musculoskeletal disorders. This cytokine activation can be attributed to the SARS-CoV-2 spike protein, other components of the vaccine, or the adenoviral vector used^67,68^.

New-onset autoimmune manifestations, including Guillain-Barré syndrome (GBS), rheumatoid arthritis, and systemic lupus erythematosus, have been reported in eleven cases following COVID-19 vaccination, particularly after the first dose. The precise nature of the link between the COVID-19 vaccine and autoimmune symptoms is still unclear, whether it is coincidental or causal. Molecular mimicry, the generation of specific autoantibodies, and the influence of specific vaccination adjuvants are all thought to play a role in the development of autoimmune diseases^63,69^. For instance, we documented one case of GBS, a rare autoimmune neurological disorder that affects the peripheral nerves and nerve roots. GBS has been associated with other vaccines such as rabies, hepatitis A and B, influenza, and more recently, the COVID-19 vaccine^70,71^.

In this study, we documented the occurrence of symptoms suggesting vaccine-induced myocarditis and pericarditis, including chest pain (88 cases), shortness of breath (103 cases), and sensations of a fast-beating, fluttering, or pounding heart (34 cases). These presentations align with the CDC report on these conditions^72^. Our findings are consistent with previous research indicating that COVID-19 vaccine-related myocarditis primarily affects young men and is more commonly associated with mRNA vaccines such as those developed by Pfizer-BioNTech and Moderna^73^.

We observed a statistically significant difference in the occurrence of serious adverse events (AEs) among different vaccine types. We identified 10 cases of VITT out of 3,239 vaccine doses, which is a rare syndrome involving venous or arterial thrombosis at unusual sites such as cerebral venous thrombosis (CVT) and splenic thrombosis. Additionally, we found 10 cases of thrombosis out of 3,239 vaccine doses, a comparable rate to reports from the US (17 cases of VITT, 14 cases of thrombosis out of 7,000 participants after the J&J vaccine) and lower than the European Medicines Agency (EMA) (222 cases of thrombosis out of 35 million participants after the AstraZeneca vaccine)^74,75^. VITT occurs when DNA leaks from the imperfect adenoviral vector used in AstraZeneca and J&J vaccines, infects cells, binds to platelet factor 4 (PF4), and triggers the production of anti-PF4 autoantibodies^76^.

We also discovered a significant increase in post-vaccination COVID-19 cases among individuals previously infected with COVID-19. Such findings may raise the issue of the benefit of vaccines for people who were previously infected with SARS-CoV-2. It is noteworthy that a study conducted in Kentucky (May–June 2021), reported an odds ratio of 2.34 (95% CI 1.58–3.47) of re-infection among unvaccinated participants compared to those who were fully vaccinated, suggesting that full vaccinations after a past SARS-CoV-2 infection provide additional protection by decreasing its transmissibility by shortening the duration of infectivity and so decrease the transmissibility^77^. Therefore, vaccination should be offered to all eligible individuals regardless of their previous infection status. While there is limited epidemiological evidence supporting the benefits of vaccination for previously infected individuals, our study supports the notion.

Regarding the frequency of post-vaccination COVID-19 in relation to the number of doses, the interpretation of the increase in infections after the second dose is still uncertain. Cumulatively, they were part of the sample that received the first dose, resulting in a significantly lower difference. Notably, the second dose can cause up to a tenfold increase in antibody levels, a stronger T-cell response, as well as more changes in the immune cells. Moreover, multiple variants of SARS-CoV-2 have emerged, primarily focused on the spike protein, a crucial element for developing vaccine candidates. Diverse vaccinations are currently undergoing clinical trials and demonstrating remarkable outcomes, however, their effectiveness still requires evaluation in various SARS-CoV-2 variants^4,20^.

### Strengthens and Limitations:

We carried out a multicenter study in six Arab countries that included the assessment of AEs associated with eight different vaccine types. We were able to identify several associated factors with post-vaccination AEs, which can aid in monitoring and follow-up efforts during and after vaccination campaigns. Additionally, our study included patients from a previous wave of COVID-19, allowing us to track AEs across different vaccine doses. However, it is important to acknowledge the limitations of our study. Firstly, being an observational study, it is susceptible to bias and confounding issues. Secondly, the use of an online self-administered survey introduces limitations such as data accuracy concerns due to recall bias, sampling bias (as more than 80% of participants were well-educated), and availability bias (excluding individuals who couldn't access or use the Internet, and those who were illiterate or deceased). Thus, our study population may not represent the entire population. Furthermore, assessing SARS-CoV-2 infection rates after vaccination is complicated by the presence of the delta variant and other variants of concern, especially as the immunity from previous vaccinations may be waning. The timing between the first and second doses is relatively close together, but the interval between the second and third doses can vary widely across countries. The availability of COVID-19 confirmatory testing in the studied countries also affects the diagnosis of infection rates, potentially missing asymptomatic cases. Another limitation is the lack of assessment of participants' pre-COVID-19 vaccine health status, making it challenging to differentiate pre-existing health issues from those related to the COVID-19 vaccine. The use of a reporting system for the participants to report the AEs themselves can introduce bias in exaggerating or underreporting some AEs. Although these limitations exist, our findings are consistent with those of other international studies. Lastly, the variation in response rate among countries with a low number of responses in some e.g. Syria may be due to the method of sample collection using an online questionnaire, compounded by political unrest in some countries (e.g. Syria) hindering internet access. It is important to interpret the data of vaccine and AE rates while considering such political conditions for further extensive studies. Such variation can affect the generalizability and comparisons of results among such countries.

## Conclusions

In conclusion, with the lack of multicenter or extensive studies in the Arab countries, the present multicenter study contributes to the literature regarding AEs of the COVID-19 vaccine during the fourth wave of the pandemic. The most frequently used vaccine type was Pfizer-BioNTech, followed by AstraZeneca and Sinovac. More than three-fourths of participants reported AEs after the first dose. In line with most previous studies, the majority was mild in severity and local in nature as injection site pain and redness. Different vaccines showed variable rates of AEs following the different doses. AEs were more frequently reported among older individuals, women, and those with a history of previous COVID-19 infection. Further extensive, thorough, and generalizable research is needed to draw solid conclusions.

## Recommendations:

Based on our findings, we recommend.

- Conducting more prospective cohort studies with a larger sample size per country to investigate the frequency and mechanisms of various AEs following vaccination.
- Clinical trials should prioritize the study of serious adverse events such as thrombosis, menstrual cycle abnormalities, and blood pressure changes.
- More attention should be directed toward countries with political conditions that hinder the vaccination process while cautiously interpreting their current results.
- Public awareness regarding vaccine side effects should be raised, and tailored health education messages should be designed according to the demographic and social characteristics of the target population.
- Implementation of a nationwide ongoing safety evaluation is crucial, particularly in monitoring and understanding the occurrence of unusual vaccine-related adverse effects with ensuring accessibility and availability of necessary services at vaccination centers is important.
- Reporting any health problems experienced after vaccination to the VAERS is encouraged, and healthcare providers should refer to clinical considerations for further clinical advice and recommendations.
- Additionally, healthcare providers should be aware of these potential adverse events and provide appropriate guidance and support to individuals receiving the vaccine.

## Methods

### Study design and participants

The study utilized a cross-sectional survey design and involved a total of 1,564 vaccinated participants from six Arab countries, namely Egypt, Saudi Arabia, Syria, Libya, Iraq, and Algeria. To be included, the individual had to be citizens or residents of one of the six Arab countries, have received COVID-19 vaccination, and are older than 12 years. Unvaccinated participants, participants who couldn't access, use, or deal with the online platform or smart devices, illiterate participants, and participants with complicated medical, mental, or psychological disorders were excluded from the study. The study adhered to the Strengthening The Reporting of Observational Studies in Epidemiology (STROBE) Checklist in its entirety^42^ (online Appendix 1).

### Sample size and sampling techniques

The sample size was calculated using Epi Info statistical calculator 7.2.5. version, which is a trademark of the Centers for Disease Control and Prevention (CDC), with the following parameters: a confidence interval of 95%, an expected frequency of 50%, and an acceptable margin of error of 5%. The reported adverse events rate after COVID-19 vaccination ranged widely between 13 and 90%^12,24–26^. We adopted a conservative estimate of 50% for the prevalence parameter in calculating the sample size. The minimum sample size was 400 responses. The sample size was increased to 1564 to increase the power of the study.

A multistage sampling technique was employed in each country, involving the random selection of three governorates from each country, followed by the selection of one urban area and one rural area from each governorate. The number of samples obtained from each area was based on the vaccination coverage and the fulfillment of the selection criteria. Because the data available for the probable geographical variations in vaccination coverage and the rate of people taking booster doses are only from a few countries and are skewed, we relied on the data of first doses (percentages of the population vaccinated with the first dose) to have a representative sample. The context of sampling and vaccination in the included countries is shown in online Appendix 2.

### Data collection

#### Data collection tool

A questionnaire was developed based on the existing literature^7,9,12–15^ and underwent a rigorous translation process. The questionnaire was initially created in English, and translated into Arabic by a bilingual panel of two healthcare professionals and one qualified medical translator. The back translation for accuracy was approved by two English-speaking translators, and the original panel was consulted in case there were any issues.

To validate the content of the survey, six family medicine and six public health and community medicine experts, two from each country, were invited to fill in the survey and assess the clarity, comprehension, and relevance of the questionnaire. We adjusted the questionnaire to ensure both relevance and feasibility among our population according to the experts’ comments. Afterward, a pilot study was conducted involving 30 vaccinated participants from each country^43–45^. Additionally, the reliability and internal consistency of the survey were assessed using Cronbach’s alpha which was 0.78 which was deemed acceptable.

The questionnaire consisted of two main sections. The first was for sociodemographic data, health-related factors, and history of SARS-CoV-2 infection. Secondly, COVID-19 vaccination-related data included vaccine type and doses, vaccination setting, self-reported AEs, and rate of SARS-CoV-2 infection after vaccination. The study questionnaire is available in online Appendix 3.

#### Data collection process

The data collection process took place in the period between January 1st and January 22nd, 2022. In line with VAERS system^18,19^, the questionnaire was administered through online platforms including websites and social media such as Facebook, Twitter, official emails, and WhatsApp groups. In addition, a community-based sample from various public places (such as schools, mosques, malls, and educational settings) responded to the questionnaire using either tablets or smartphones provided by the data collectors or by scanning the QR code, especially for children less than 16 years old after informed written consent from their parents. All questions were obligatory to avoid incomplete forms. Only a single answer was allowed per each logging email to avoid duplicate responses. Participants completed and submitted the questionnaire after providing their consent to participate. Follow-up messages and reminders were sent to increase the response rate.

### Study outcomes

The primary outcome was the reporting of AEs following COVID-19 vaccination. A total of 32 self-reported COVID-19 vaccine-related AEs were considered and categorized into no local/general AEs, local AEs at the injection site, and general AEs (systemic and serious AEs) based on CDC guidance. Secondary outcomes were factors associated with AEs after the first dose and the association between pre-vaccination COVID-19 infection and post-vaccination COVID-19.

### Statistical analysis

The collected data was coded into SPSS version 27 for analysis. The normally distributed quantitative data was presented as mean ± SD, after testing by the Kolmogorov–Smirnov test. Analysis of variance (ANOVA) and post-hoc tests were used to analyze normally distributed quantitative data. Qualitative data such as age groups and sex were presented as frequency and percentage, and the chi-squared test (χ2), and Fisher’s exact test were used to test the association between categorical variables. A simple logistic regression analysis within the framework of a generalized linear model technique was used to examine the association of each potential factor with the binary outcome of vaccination adverse effects (no AE, and with AEs). Independent variables included baseline variables such as age, sex, comorbidities, and nationalities. Next, we fitted a final logistic regression model using a stepwise method to examine the independent associations of each potential factor with the outcome of interest. In the stepwise regression method, first, we added into the model all those factors that were significant (p < 0.05) in the univariable analyses. Then we retained significant (p < 0.05) factors in the model and iteratively tested all non-significant variables in the final model for possible significance in the subsequent steps. We used likelihood ratio tests to examine the statistical significance of each factor. From the above fitted final model, we estimated and reported the adjusted odds ratios (OR) with a 95% confidence interval (CI). To examine the final model fit, we used the Hosmer–Lemeshow test. The level of significance was set at (p < 0.05).

### Ethical considerations

The study was conducted in accordance with the Declaration of Helsinki and approved by the Institutional Review Board (or Ethics Committee) of the research center at Zagazig University (IRP#9288–25-1-2022a). Participants were provided with informed consent before answering the questionnaire. The questionnaire did not include sensitive or private questions, and respondents' identities remained anonymous.

## Institutional review board statement

The study was conducted in accordance with the Declaration of Helsinki, and approved by the Institutional Review Board (or Ethics Committee) of the research center at Zagazig University IRP#9288–25-1-2022a.

## Informed consent statement

Participants were provided with informed consent before answering the questionnaire. The questionnaire did not include sensitive or private questions, and respondents' identities remained anonymous.

### Supplementary Information

Supplementary Information.

## Data Availability

The datasets used and/or analyzed during the current study are available from the corresponding author upon reasonable request.
