# Supplementary material for: Exploring the reported adverse effects of COVID-19 vaccines among vaccinated Arab populations: a multi-national survey study
Source: Sci Rep. 2024 Feb 27;14:4785. doi: 10.1038/s41598-024-54886-0 (PMC10899622; doi:10.1038/s41598-024-54886-0)
Supplement: Supplementary file 1 — Supplementary Information. [file 41598_2024_54886_MOESM1_ESM.pdf]

Exploring the Reported Adverse Effects of COVID-19 Vaccines among Vaccinated Arab Populations: A Multi-National Survey Study

Appendix Files

Appendix 1: STROBE Statement: checklist of items that should be included in reports of observational studies

	Item No.	Recommendation	Page No.
**Title and abstract**	1	(*a*) Indicate the study's design with a commonly used term in the title or the abstract	**1**
		(*b*) Provide in the abstract an informative and balanced summary of what was done and what was found	**2**
**Introduction**			
Background/rationale	2	Explain the scientific background and rationale for the investigation being reported	**3-4**
Objectives	3	State specific objectives, including any prespecified hypotheses	**4**
**Methods**			
Study design	4	Present key elements of study design early in the paper	**11**
Setting	5	Describe the setting, locations, and relevant dates, including periods of recruitment, exposure, follow-up, and data collection	**11**
Participants	6	(*a*) *Cohort study*—Give the eligibility criteria, and the sources and methods of selection of participants. Describe methods of follow-up<br>*Case-control study*—Give the eligibility criteria, and the sources and methods of case ascertainment and control selection. Give the rationale for the choice of cases and controls<br>*Cross-sectional study*—Give the eligibility criteria, and the sources and methods of selection of participants	**11**
		(*b*) *Cohort study*—For matched studies, give matching criteria and number of exposed and unexposed<br>*Case-control study*—For matched studies, give matching criteria and the number of controls per case	
Variables	7	Clearly define all outcomes, exposures, predictors, potential confounders, and effect modifiers. Give diagnostic criteria, if applicable	**11-12**
Data sources/	8*	For each variable of interest, give sources of data and details of methods of assessment (measurement). Describe	**11**

measurement		comparability of assessment methods if there is more than one group	
Bias	9	Describe any efforts to address potential sources of bias	**11-12**
Study size	10	Explain how the study size was arrived at	**11**

Quantitative variables	11	Explain how quantitative variables were handled in the analyses. If applicable, describe which groupings were chosen and why	**12**
Statistical methods	12	(*a*) Describe all statistical methods, including those used to control for confounding	**12**
		(*b*) Describe any methods used to examine subgroups and interactions	**12**
		(*c*) Explain how missing data were addressed	**12**
		(*d*) *Cohort study*—If applicable, explain how loss to follow-up was addressed<br>*Case-control study*—If applicable, explain how matching of cases and controls was addressed<br>*Cross-sectional study*—If applicable, describe analytical methods taking account of sampling strategy	**12**
		(*e*) Describe any sensitivity analyses	**-**
**Results**			
Participants	13*	(a) Report numbers of individuals at each stage of study—eg numbers potentially eligible, examined for eligibility, confirmed eligible, included in the study, completing follow-up, and analysed	**5**
		(b) Give reasons for non-participation at each stage	**-**
		(c) Consider use of a flow diagram	**-**
Descriptive data	14*	(a) Give characteristics of study participants (eg demographic, clinical, social) and information on exposures and potential confounders	**5**
		(b) Indicate number of participants with missing data for each variable of interest	-
		(c) *Cohort study*—Summarise follow-up time (eg, average and total amount)	
Outcome data	15*	*Cohort study*—Report numbers of outcome events or summary measures over time	
		Case-control study—Report numbers in each exposure category, or summary measures of exposure	
		Cross-sectional study—Report numbers of outcome events or summary measures	**6**
Main results	16	(*a*) Give unadjusted estimates and, if applicable, confounder-adjusted estimates and their precision (eg, 95% confidence interval). Make clear which confounders were adjusted for and why they were included	**6**
		(*b*) Report category boundaries when continuous variables were categorized	
		(*c*) If relevant, consider translating estimates of relative risk into absolute risk for a meaningful time period	

Continued on next page

Other analyses	17	Report other analyses done—eg analyses of subgroups and interactions, and sensitivity analyses	**5-6**
**Discussion**			
Key results	18	Summarise key results with reference to study objectives	**7-8**
Limitations	19	Discuss limitations of the study, taking into account sources of potential bias or imprecision. Discuss both direction and magnitude of any potential bias	**9**
Interpretation	20	Give a cautious overall interpretation of results considering objectives, limitations, multiplicity of analyses, results from similar studies, and other relevant evidence	**7-8**
Generalisability	21	Discuss the generalisability (external validity) of the study results	**9**
**Other information**			
Funding	22	Give the source of funding and the role of the funders for the present study and, if applicable, for the original study on which the present article is based	**13**

*Give information separately for cases and controls in case-control studies and, if applicable, for exposed and unexposed groups in cohort and cross-sectional studies.

Note: An Explanation and Elaboration article discusses each checklist item and gives methodological background and published examples of transparent reporting. The STROBE checklist is best used in conjunction with this article (freely available on the Web sites of PLoS Medicine at http://www.plosmedicine.org/, Annals of Internal Medicine at http://www.annals.org/, and Epidemiology at http://www.epidem.com/). Information on the STROBE Initiative is available at www.strobe-statement.org.

Appendix 2: Context of sampling and vaccination process

By 1st of January 2022	Total Population	Infected	1st dose	2nd dose	Booster dose	Types of vaccines	Sample size
**Saudi Arabia (SA)**	35,683,504	590,871	25,501,361 (71.5%)	23,647,878 (91.9%)	Compulsory by 1st February 2022	J&J, Astra-Zeneca, Pfizer and Moderna	444 (28.45)
**Egypt**	118,600,000	384,728	35,345,794 (29.8%)	22,829,583 (19.25%)	Started among HCPs, and risky groups after 6 months from the 2nd dose	J&J , Astra-Zeneca, Pfizer, Moderna, Sputnik V, Sputnik -Light, Sinovac, Sinopharm	345 (22.0%)
**Libya**	7,018,138	389,650	1, 872, 292 (26.7%)	851,595 (12.1 %)	Not started	Astra-Zeneca, Sputnik V, Sputnik –Light, Sinopharm and Pfizer	218 (14.0%)
**Algeria**	45,106,964	218,037	7,245,900 (16.1%)	5,786,000 (12.82%)	Started 18 December 2021	Astra-Zeneca, Sputnik V and Sputnik -Light,	213 (13.0%)
**Iraqi**	41,685,691	2,093,740	8 735 048 (21%)	5,801,589 (13.9%)	Not started yet	Astra-Zeneca, Sputnik V, Sputnik -Light, Sinovac and Sinopharm.	183 (11.7)
**Syria**	18,300,000	2,474,778	2,835,406 (15.5%)	50,205 (0.3%)	Not started yet	Astra-Zeneca, Sputnik V, Sputnik –Light and Sinopharm	124 (7.9%)

Appendix 3: The study questionnaire in English and Arabic languages.

الأعراض الجانبية للتحصين ضد فيروس كرونا المستجد بعد
الجرعات المختلفة ومحدداتها بين المحصنين أثناء الموجة الرابعة لوباء الكرونا

((Side effects of vaccination against the emerging coronavirus after different doses and their determinants among those vaccinated during the fourth wave of the coronavirus pandemic))

الموافقة المستنيرة:

نحن مجموعة من الاطباء الباحثين ، تستهدف هذه الدراسة كل الفئات المحصنه لدراسة الأعراض الجانبية للتحصين ضد فيروس كرونا المستجد بعد الجرعات المختلفة علما بان هذه البيانات سرية ،لن تستخدم الا لأغراض بحثية ،في حال وجود اي استفسار الرجاء التواصل مع الباحث الرئيسي.

Informed Consent:

((*We are a group of research doctors. This study targets all immunized groups to study the side effects of vaccination against the emerging coronavirus after different doses. Note that this data is confidential and will only be used for research purposes. If you have any questions, please contact the principal researcher.*))

amers@moh.gov.sa

1.هل توافق علي المشاركة:

((1.Do you agree to participate:))

نعم ☐ لا ☐

((*yes*)) ((*No*))

2.هل تلقيت اللقاح ضد فيروس كورونا المستجد :

((2.Have you received the vaccine against the emerging coronavirus?))

☐ لا (No)

☐ نعم بشكل اختياري (Yes, voluntary)

☐ نعم بشكل اجباري للعمل،السفر، أو التعليم (Yes, compulsorily for work, travel, or school…)

---

3. العمر بعدد السنوات :

((*3.Age in years :*))

☐ من 45 الي اقل من 60 ((From 45 to less than 60))	☐ من 30 الي اقل من 45 ((From 30 to less than 45))	☐ من 18الي اقل من 30 ((From 18 to less than 30))	☐ من 12الي اقل من 18 ((From 12 to less than 18))
		☐ 75 عام فاكثر ((75 years or more))	☐ من 60 الي اقل من 75 ((From 60 to less than 75))

4.الجنس

((4.Sex))

☐ Female انثي. ☐ Male ذكر

.5 مستوي التعليم

((5.Educational level))

تعليم ابتدائي / يقرأ ويكتب ☐<br>((Primary education/ reads and writes))	ثانوي / تعليم متوسط ☐<br>((Middle/secondary education))	دراسات عليا / عليم جامعي ☐<br>((University education/ postgraduate studies))

.6 الوظيفة الحالية :

((6.current occupation:))

طالب / ربة منزل / لا يعمل ☐<br>((Not working/housewife/student))	يعمل في المجال الطبي ☐<br>((works in healthcare))	يعمل في مجالات أخرى ☐<br>((others))

.7 محل السكن:

((7.Current residence))

داخل المدينة (حضر) ☐<br>((Within the city (urban)))	خارج المدينة (ريف) ☐<br>((Out of city (rural)))

.8 الوزن بالكيلو: ________ **((8.Weight in kilograms))**

.9 الطول بالمتر: ________ **((9.Height in meters))**

10 - الجنسية :

((10.nationality))

ليبي.((Libyan)) ☐	سوري((Syrian)) ☐
مصري((Egyptian)) ☐	عراقي((Iraqi)) ☐
سعودي((Saudi)) ☐	اخرى ((others)) ☐
جزائري((Algerian)) ☐	

.11 هل تعاني من امراض مزمنة؟

((11.Do you have any chronic illness?))

لا ☐<br>((No))
مرض عضوي... كالضغط او السكر.....الخ ☐<br>((Organic illness like hypertension,diabetes,..etc))
مرض نفسي عصبي ... كالقلق او الاكتئاب ....الخ. ☐<br>((psychological illness like anxiety, depression,....etc))

12. هل تتلقى احدي هذه العلاجات:

((12.Do you take treatment for one Or more of the following:))

علاج هرموني ☐((hormonal therapy))
علاج احد الأمراض المناعية ((immunity therapy)) ☐
كورتيزون أو احد الادوية المثبطة بالمناعة. ((Cortisone or an immunosuppressive drug.))☐
مكملات غذائية. ((dietary supplement)) ☐
أدوية لسيولة الدم ((blood thinning medications)) ☐

13 . هل اصبت بعدوي (كوفيد١٩) قبل اللقاح:
((13.have you had (Covid-19) infection before the vaccine))

لا ☐ ((no))
نعم... إصابة من دون اعراض ☐ ((Yes... an infection without symptoms))
نعم...اصابة باعراض وتم علاجي في المنزل ☐ ((Yes...I had symptoms and was treated at home))
نعم...اصابه بأعراض وتم علاجي في المستشفى ☐ ((Yes...I had symptoms and was treated in the hospital))
اشتباه.. (فقدان شم، تذوق, حرارة) من دون فحص او عينة سلبية. ☐ ((Suspicion... (loss of smell, taste, fever) without medical examination or a negative test))

14.اين تلقيت اللقاح ضد فيروس كرونا المستجد:

((14.Where did you receive the vaccine against the emerging coronavirus?))

في حملة متنقلة في.. (نادي، مول، العمل.. الخ) ☐ ((On a mobile campaign ...(club, mall, work, etc.)))
في مركز رعاية صحية أولية ☐ ((In a primary health care center))
في مستشفي او مركز لقاحات ☐ ((In a hospital or vaccination center))

15. اذكر نوع اللقاح الذي اخذته في الجرعة الأولى:<br>((15.Choose the type of vaccine you took in the first dose))				
سيوفارم ☐<br>((Sinopharm))	فايزر ☐<br>((Pfizer))	استرازينيكا ☐<br>((astrazeneca))	جونسون اند جونسون ☐<br>((The Johnson & Johnson (Janssen)))	مودرنا ☐<br>((Moderna))
اخري<br>((others))	سينوفاك ☐<br>((Sinovec))	سيبوتنيك في ☐<br>((Sputnik V))	سيبوتيك لايت ☐<br>((Sputnik light))	لا اعرف النوع ☐<br>((I don't know the type))

16. الأعراض الجانبية بعد الجرعه الأولي (أختر كل ما تراه مناسبا) (مسموح بأكثر من إجابة) :

((16.Side effects after the first dose (Choose all you deem appropriate (More than one answer is allowed))))

لا يوجد ((none))	
ألام / ثقل / احمرار/ورم في الذراع ((Pain/heaviness/redness/swelling in the arm))	☐
غثيان او قيء ((Nausea or vomiting))	☐
تكسير في الجسم ((body aches))	☐
آلام العظام و العضلات ((Bone and muscle pain))	☐
خمول وارهاق عام ((Lethargy and general fatigue))	☐
(الحساسية المفرطة) علي شكل طفح جلدي /التهاب بالاغشية المخاطية المبطنة للانف او الأذن<br>(((Hypersensitivity) in the form of a skin rash/inflammation of the mucous membranes lining the nose or ear))	☐
صداع ((headache))	☐
تشنجات او صرع ((Convulsions or epilepsy))	☐
سيلان .او احتقان في الانف ((runny nose. Or nasal congestion))	☐
احتباس السوائل بالجسم) بمنطقة الجفون -القدمين -البطن)<br>((Fluid retention in the body (in the eyelids, feet, and abdomen).))	☐
حدوث جلطات ((abnormal clot formation))	☐
انخفاض الصفائح الدموية ((low platelet count))	☐
آلام في الصدر ((chest pain))	☐
صعوبات في التنفس ((difficulty breathing))	☐
آلام مستمرة في المعدة ((prolonged abdominal pain))	☐
اضطرابات في الرؤية ((visual disturbances))	☐
التهابات في عضلة القلب -زياده ، اضطرابات بضربات القلب<br>((Inflammation of the heart muscle -increased heart rate, disturbances in heart rhythm))	☐
تغيير في طبيعة الدورة الشهرية (انقطاعها /تغيير في الكمي / اضطرابها)<br>(((Change in the nature of the menstrual cycle) its cessation / change in quantity / disturbance))	☐
ظهور او تورم العقد الليمفاوية ((The appearance or swelling of lymph nodes))	☐
اضطرابات نفسية او عصبية ((Psychological or nervous disorders))	☐
اضطرابات بضغط الدم ((Blood pressure disorders))	☐
اضطرابات وظائف الكبد ((Liver function disorders))	☐
اضطرابات وظائف الكلي /التهاب الكلي /بول رغوي<br>((Kidney function disorders/nephritis/frothy urine))	☐
ظهور امراض او اضطرابات بالمناعة ((The appearance of diseases or immune disorders))	☐

سقوط في الشعر -او ظهور شعر ابيض بمعدل اكثر من الطبيعي<br>((Hair loss - or the appearance of white hair at a rate greater than normal))	☐
مشاكل في الجلد /ظهور بقع بيضاء /كدمات زرقاء او بقع حمراء<br>((Skin problems / appearance of white spots / blue bruises or red spots))	☐
(في حال كنت حامل) حدث اجهاض / ولادة مبكره /اي مضاعفات اخري<br>((If you are pregnant (miscarriage / premature birth / any other complications).))	☐
(في حال كنت مرضع). هل تاثرت الرضاعة او الرضيع<br>(((If you are breastfeeding). Has breastfeeding and the baby been affected?))	☐
(متلازمة جيلان باري(ضعف عضلي سريع) او اضرابات التهابات في الجهاز العصبي (المخ, الغشاء السحائي)<br>((Guillain-Barré syndrome (rapid muscle weakness) or inflammatory disorders of the nervous system (brain, meningeal membrane)))	☐
تأثر حاسة السمع ((hearing affection))	☐
فقدان حاسة الشم او التذوق ((loss of smell or taste))	☐
اضطرابات في النوم ((sleep disturbances))	☐
في حال وجود أعراض أخرى أذكرها ((if you experienced other symptoms, please mention them))	

17. كيف تعاملت مع مضاعفات الجرعة الاولي (مسموح بأكثر من إجابة):

((17. How did you deal with complications from the first dose (more than one answer is allowed):))

☐ لا شيء<br>((none))	☐ خافضات الحرارة ومسكنات الألم<br>((painkillers and antipyretics))	☐ إستشارة طبية<br>((I sought medical consultation))	☐ تم حجزي بأحد المستشفيات<br>((I was admitted to the hospital))

18. هل اصبت بعدوي الكرونا بعد الجرعة الاولي للتطعيم :

((18.have you had COVID-19 infection after first dose of the vaccine:))

لا ((no))	☐
نعم ... بدون اعراض ((Yes...without symptoms))	☐
نعم.......اصبت باعراض وتم علاجي في المنزل<br>((Yes.......I had symptoms and was treated at home))	☐
نعم.. اصبت باعراض وتم علاجي في المستشفى<br>((Yes. ..I had symptoms and was treated in the hospital))	☐

.19 اذكر نوع اللقاح الذي اخذته في الجرعة الثانية:<br>((19.Choose the type of vaccine you took in the second dose))				
سيوفارم ☐<br>((Sinopharm))	فايزر☐<br>((Pfizer))	استرازينيكا☐<br>((astrazeneca))	جونسون اند جونسون ☐<br>((The Johnson & Johnson (Janssen)))	مودرنا ☐<br>((Moderna))
اخري<br>((others))	سينوفاك☐<br>((Sinovec))	سيبوتنيك في☐<br>((Sputnik V))	سيبوتيك لايت ☐<br>((Sputnik light))	لا اعرف النوع ☐<br>((I don’t know the type))

.20 الأعراض الجانبية بعد الجرعه الثانية (أختر كل ما تراه مناسبا) (مسموح بأكثر من إجابة) :

((20.Side effects after the second dose (Choose all you deem appropriate (More than one answer is allowed))))

لا يوجد ((none))	
ألام /ثقل /احمرار/ورم في الذراع ((Pain/heaviness/redness/swelling in the arm))	☐
غثيان او قيء ((Nausea or vomiting))	☐
تكسير في الجسم ((body aches))	☐
آلام العظام و العضلات ((Bone and muscle pain))	☐
خمول وارهاق عام ((Lethargy and general fatigue))	☐
(الحساسية المفرطة) علي شكل طفح جلدي /التهاب بالاغشية المخاطية المبطنة للانف او الأذن<br>(((Hypersensitivity) in the form of a skin rash/inflammation of the mucous membranes lining the nose or ear))	☐
صداع ((headache))	☐
تشنجات او صرع ((Convulsions or epilepsy))	☐
سيلان .او احتقان في الانف ((runny nose. Or nasal congestion))	☐
(احتباس السوائل بالجسم) بمنطقة الجفون -القدمين -البطن<br>((Fluid retention in the body (in the eyelids, feet, and abdomen).))	☐
حدوث جلطات ((abnormal clot formation))	☐
انخفاض الصفائح الدموية ((low platelet count))	☐
آلام في الصدر ((chest pain))	☐
صعوبات في التنفس ((difficulty breathing))	☐
آلام مستمرة في المعدة ((prolonged abdominal pain))	☐
اضطرابات في الرؤية ((visual disturbances))	☐
التهابات في عضلة القلب -زياده ، اضطرابات بضربات القلب<br>((Inflammation of the heart muscle -increased heart rate, disturbances in heart rhythm))	☐
تغيير في طبيعة الدورة الشهرية (انقطاعها/ تغيير في الكمي/ اضطرابها)<br>(((Change in the nature of the menstrual cycle) its cessation / change in quantity / disturbance))	☐
ظهور او تورم العقد الليمفاوية ((The appearance or swelling of lymph nodes))	☐
اضطرابات نفسية او عصبية ((Psychological or nervous disorders))	☐
اضطرابات بضغط الدم ((Blood pressure disorders))	☐
اضطرابات وظائف الكبد ((Liver function disorders))	☐
اضطرابات وظائف الكلي /التهاب الكلي /بول رغوي<br>((Kidney function disorders/nephritis/frothy urine))	☐
ظهور امراض او اضطرابات بالمناعة ((The appearance of diseases or immune disorders))	☐

سقوط في الشعر -او ظهور شعر ابيض بمعدل اكثر من الطبيعي<br>((Hair loss - or the appearance of white hair at a rate greater than normal))	☐
مشاكل في الجلد /ظهور بقع بيضاء /كدمات زرقاء او بقع حمراء<br>((Skin problems / appearance of white spots / blue bruises or red spots))	☐
(في حال كنت حامل) حدث اجهاض / ولادة مبكره /اي مضاعفات اخري<br>((If you are pregnant (miscarriage / premature birth / any other complications).))	☐
(في حال كنت مرضع). هل تاثرت الرضاعة او الرضيع<br>(((If you are breastfeeding). Has breastfeeding and the baby been affected?))	☐
(متلازمة جيلان باري) ضعف عضلي سريع) او اضرابات التهابات في الجهاز العصبي (المخ, الغشاء السحائي)<br>((Guillain-Barré syndrome (rapid muscle weakness) or inflammatory disorders of the nervous system (brain, meningeal membrane)))	☐
تأثر حاسة السمع ((hearing affection))	☐
فقدان حاسة الشم او التذوق ((loss of smell or taste))	☐
اضطرابات في النوم ((sleep disturbances))	☐
في حال وجود أعراض أخرى أذكرها ((if you experienced other symptoms, please mention them))	

21. كيف تعاملت مع مضاعفات الجرعة الثانية (مسموح بأكثر من إجابة):

((21. How did you deal with complications from the second dose (more than one answer is allowed):))

☐ تم حجزي بأحد المستشفيات<br>((I was admitted to the hospital))	☐ إستشارة طبية<br>((I sought medical consultation))	☐ خافضات الحرارة ومسكنات الألم<br>((painkillers and antipyretics))	☐ لا شيء<br>((none))

22. هل اصبت بعدوي الكرونا بعد الجرعة الثانية للتطعيم :

((22.have you had COVID-19 infection after second dose of the vaccine:))

لا ((no))	☐
نعم ... بدون اعراض ((Yes...without symptoms))	☐
نعم.......اصبت باعراض وتم علاجي في المنزل<br>((Yes.......I had symptoms and was treated at home))	☐
نعم.. اصبت باعراض وتم علاجي في المستشفى<br>((Yes. ..I had symptoms and was treated in the hospital))	☐

23. اذكر نوع اللقاح الذي اخذته في الجرعة الثالثة:<br>((23.Choose the type of vaccine you took in the third dose))				
سيوفارم ☐<br>((Sinopharm))	فايزر ☐<br>((Pfizer))	استرازينيكا ☐<br>((astrazeneca))	جونسون اند جونسون ☐<br>((The Johnson & Johnson (Janssen)))	مودرنا ☐<br>((Moderna))
اخري<br>((others))	سينوفاك ☐<br>((Sinovec))	سبيوتنيك في ☐<br>((Sputnik V))	سبيوتيك لايت ☐<br>((Sputnik light))	لا اعرف النوع ☐<br>((I don't know the type))

24. الأعراض الجانبية بعد الجرعه الثالثة (أختر كل ما تراه مناسبا) (مسموح بأكثر من إجابة) :

((24.Side effects after the third dose (Choose all you deem appropriate (More than one answer is allowed))))

لا يوجد ((none))	
ألام /ثقل /احمرار/ورم في الذراع ((Pain/heaviness/redness/swelling in the arm))	☐
غثيان او قيء ((Nausea or vomiting))	☐
تكسير في الجسم ((body aches))	☐
آلام العظام والعضلات ((Bone and muscle pain))	☐
خمول وارهاق عام ((Lethargy and general fatigue))	☐
(الحساسية المفرطة) علي شكل طفح جلدي /التهاب بالاغشية المخاطية المبطنة للانف اوالأذن<br>(((Hypersensitivity) in the form of a skin rash/inflammation of the mucous membranes lining the nose or ear))	☐
صداع ((headache))	☐
تشنجات او صرع ((Convulsions or epilepsy))	☐
سيلان .او احتقان في الانف ((runny nose. Or nasal congestion))	☐
(احتباس السوائل بالجسم) بمنطقة الجفون -القدمين -البطن<br>((Fluid retention in the body (in the eyelids, feet, and abdomen).))	☐
حدوث جلطات ((abnormal clot formation))	☐
انخفاض الصفائح الدموية ((low platelet count))	☐
آلام في الصدر ((chest pain))	☐
صعوبات في التنفس ((difficulty breathing))	☐
آلام مستمرة في المعدة ((prolonged abdominal pain))	☐
اضطرابات في الرؤية ((visual disturbances))	☐
التهابات في عضلة القلب -زياده ا، اضطرابات بضربات القلب<br>((Inflammation of the heart muscle -increased heart rate, disturbances in heart rhythm))	☐
تغيير في طبيعة الدورة الشهرية (انقطاعها /تغيير في الكمي /اضطرابها)<br>(((Change in the nature of the menstrual cycle) its cessation / change in quantity / disturbance))	☐
ظهور او تورم العقد الليمفاوية ((The appearance or swelling of lymph nodes))	☐
اضطرابات نفسية او عصبية ((Psychological or nervous disorders))	☐
اضطرابات بضغط الدم ((Blood pressure disorders))	☐
اضطرابات وظائف الكبد ((Liver function disorders))	☐
اضطرابات وظائف الكلي /التهاب الكلي /بول رغوي<br>((Kidney function disorders/nephritis/frothy urine))	☐
ظهور امراض او اضطرابات بالمناعة ((The appearance of diseases or immune disorders))	☐

☐	سقوط في الشعر -او ظهور شعر ابيض بمعدل اكثر من الطبيعي<br>((Hair loss - or the appearance of white hair at a rate greater than normal))
☐	مشاكل في الجلد /ظهور بقع بيضاء /كدمات زرقاء او بقع حمراء<br>((Skin problems / appearance of white spots / blue bruises or red spots))
☐	(في حال كنت حامل (حدث اجهاض / ولادة مبكره /اي مضاعفات اخري<br>((If you are pregnant (miscarriage / premature birth / any other complications).))
☐	(في حال كنت مرضع). هل تاثرت الرضاعة او الرضيع<br>(((If you are breastfeeding). Has breastfeeding and the baby been affected?))
☐	(متلازمة جيلان باري (ضعف عضلي سريع) او اضرابات التهابات في الجهاز العصبي (المخ, الغشاء السحائي)<br>((Guillain-Barré syndrome (rapid muscle weakness) or inflammatory disorders of the nervous system (brain, meningeal membrane)))
☐	تأثر حاسة السمع ((hearing affection))
☐	فقدان حاسة الشم او التذوق ((loss of smell or taste))
☐	اضطرابات في النوم ((sleep disturbances))
	في حال وجود أعراض أخرى أذكرها ((if you experienced other symptoms, please mention them))

25. كيف تعاملت مع مضاعفات الجرعة الثالثة (مسموح بأكثر من إجابة):

((25. How did you deal with complications from the third dose (more than one answer is allowed):))

☐ لا شيء<br>((none))	☐ خافضات الحرارة ومسكنات الألم<br>((painkillers and antipyretics))	☐ إستشارة طبية<br>((I sought medical consultation))	☐ تم حجزي بأحد المستشفيات<br>((I was admitted to the hospital))

26. هل اصبت بعدوى الكرونا بعد الجرعة الثالثة للتطعيم :

((26.have you had COVID-19 infection after third dose of the vaccine:))

☐	لا ((no))
☐	نعم ... بدون اعراض ((Yes...without symptoms))
☐	نعم.......اصبت باعراض وتم علاجي في المنزل<br>((Yes.......I had symptoms and was treated at home))
☐	نعم .. اصبت باعراض وتم علاجي في المستشفى<br>((Yes. ..I had symptoms and was treated in the hospital))

27.أي من الجرعات التي تلقيتها تعتبر الأكثر أعراض أو مضاعفات :

((27. Which of the doses you received is considered to have the most symptoms or complications:))

الاولي ((first))	☐
الثانية((second))	☐
الثالثة ((third))	☐
لايوجد ((none))	☐

شكرا لحسن تعاونكم معنا -----نتمني لكم الصحة والعافية

((Thank you for your cooperation ----- We wish you good health))
